# Transcriptional comparison of human induced and primary midbrain dopaminergic neurons

**DOI:** 10.1038/srep20270

**Published:** 2016-02-04

**Authors:** Ninuo Xia, Pengbo Zhang, Fang Fang, Zhengyuan Wang, Megan Rothstein, Benjamin Angulo, Rosaria Chiang, James Taylor, Renee A. Reijo Pera

**Affiliations:** 1 Department of Genetics; Department of Obstetrics and Gynecology; Institute for Stem Cell Biology and Regenerative Medicine, Stanford University, CA, USA; 2 Department of Cell Biology and Neurosciences, Montana State University, 207 Montana Hall, Bozeman, MT; 3 Genomic Medicine Division, Hematology Branch, NHLBI/NIH, MD 20850, USA

## Abstract

Generation of induced dopaminergic (iDA) neurons may provide a significant step forward towards cell replacement therapy for Parkinson’s disease (PD). To study and compare transcriptional programs of induced cells versus primary DA neurons is a preliminary step towards characterizing human iDA neurons. We have optimized a protocol to efficiently generate iDA neurons from human pluripotent stem cells (hPSCs). We then sequenced the transcriptomes of iDA neurons derived from 6 different hPSC lines and compared them to that of primary midbrain (mDA) neurons. We identified a small subset of genes with altered expression in derived iDA neurons from patients with Parkinson’s Disease (PD). We also observed that iDA neurons differ significantly from primary mDA neurons in global gene expression, especially in genes related to neuron maturation level. Results suggest iDA neurons from patient iPSCs could be useful for basic and translational studies, including *in vitro* modeling of PD. However, further refinement of methods of induction and maturation of neurons may better recapitulate full development of mDA neurons from hPSCs.

Parkinson’s disease (PD) is characterized by the loss of dopaminergic neurons within the *substantia nigra pars compacta*. By the time of patient death, frequently, this region of the brain has lost 50–70% of its neurons compared with the same region in unaffected individuals^1^. Midbrain dopaminergic (mDA) neurons, which constitute less than 1% of the total number of brain neurons, innervate brain regions specific for motor behavior, motivation and working memory^2^,^3^.

During development, mDA neurons arise from the midbrain-hindbrain junction and project to various forebrain targets. Transcription factors, such as OTX2 (which defines the midbrain-hindbrain boundary), play important roles during the generation of mDA neurons^4^. Later, signaling molecules sonic hedgehog (SHH), from the notochord, and Fgf8, from the isthmus, function in concert to induce floor plate dopaminergic cells in the ventral midbrain^5^. Other factors implicated in early development of mDA neurons in diverse organisms include *Wnt1, En1, En2, Nurr1, Pitx3, Lmx1B, Foxa2* and *Pax5*^6^,^7^,^8^,^9^,^10^,^11^. Ultimately, maturation of mDA neurons is marked by formation of tyrosine hydroxylase (TH) positive synaptic terminals and release of dopamine.

Under appropriate culture conditions, human embryonic stem cells (hESCs) and human induced pluripotent stem cells (hiPSCs) can be differentiated into DA neurons with midbrain characteristics^12^. A recent differentiation protocol uses BMP (Bone Morphogenic Protein) and TGFβ (transforming growth factor beta) signaling inhibitors, along with a GSK3β (Glycogen synthase kinase-3 beta) inhibitor, to directly induce floor plate DA precursors^13^. This method has been termed the “floor plate (FP)” method and demonstrates advantages over old methods in terms of reproducibility, efficiency and homogeneity. DA neurons generated by the FP strategy have been shown to engraft into rodent and primate PD models, which hold great potential for future human transplantation therapy for PD patients.

Although the generation of induced dopaminergic (iDA) neurons may provide a significant step forward towards basic and translational studies, including cell replacement therapy for PD, potential risks remain in using iPSC-derived cells. For example, some iPSCs retain epigenetic memories of their donor tissues^14^. Moreover, a recent study in mice showed that iPSC-derived Pitx3-positive iDA neurons were hyper-methylated and exhibited limited expression of developmental genes^15^. Here, we sought to compare the characteristics of induced versus primary human DA neurons. For this purpose, we employed whole genome analysis of iDA neurons by RNA-seq and microarray, then compared the results to published midbrain DA neuron gene expression data set GSE7621, which used microarrays to study gene expression of human substantia nigra dissected from controls and PD patients^16^.

## Results

### Differentiation of human pluripotent stem cells into iDA neurons

**Figure 1:**
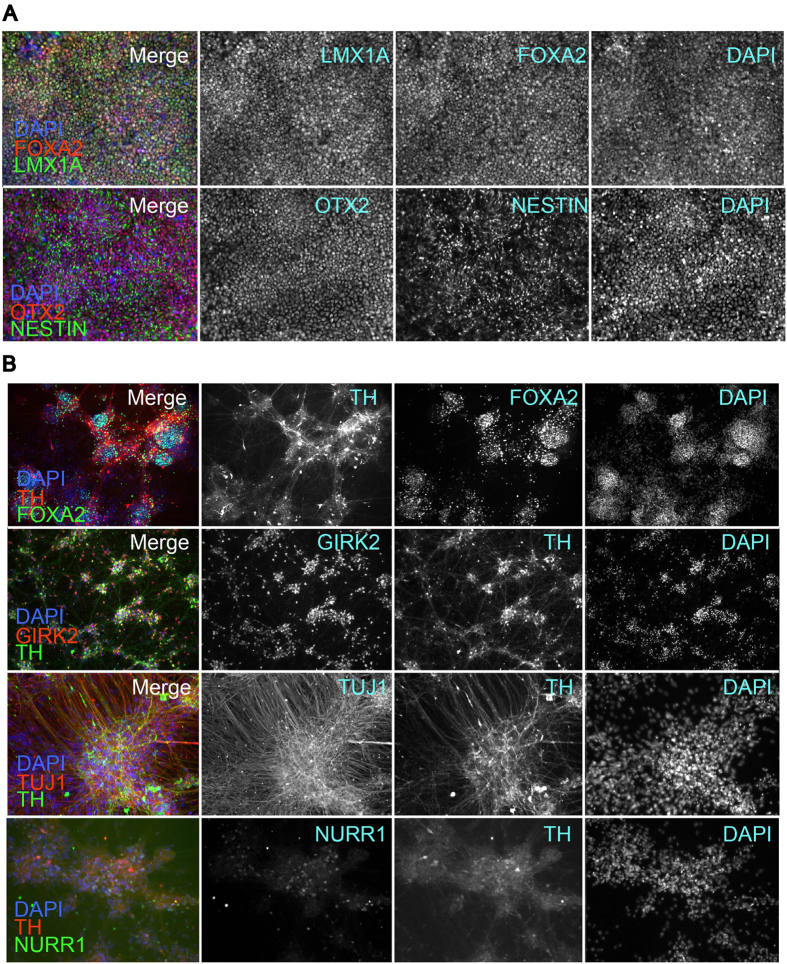
*In Vitro* differentiation of dopaminergic neurons. (**A**) Cells express floor plate markers FOXA2, LMX1a, NESTIN and OTX2 at day 11 of *in vitro* differentiation. (**B**) At day 50 of *in vitro* differentiation, cells express mature A9 dopaminergic neuron proteins TH, GIRK2, NURR1 and mature neuron marker TUJ1.

We differentiated iDA neurons from six human pluripotent stem cell (hPSC) lines: two hESC lines (H1 and H9), one control iPSC line (HUF1) and three PD patient-derived iPSC lines (HUF6, 1588, and 27760). hPSCs were induced towards dopaminergic cell fate using the FP method as described^17^ with minor modifications (materials and methods). At day 11 of differentiation, midbrain progenitor markers LMX1A, FOXA2, NESTIN and OTX2 were detected (Fig. 1A). At day 20, cells were plated onto polyorithine, fibronectin and laminin plates and cultured for another 30 days. At day 50, we detected mature DA neuron markers such as TH, GIRK2, NURR1 and TUJ1 (Fig. 1B). Also we detected a substantial increase in dopamine concentration in DA neuron growth medium analyzed by mass spectrometry (Supplemental Fig. 1B).

### Gene expression profiling reveals differences between PD and control iDA neurons

**Figure 2:**
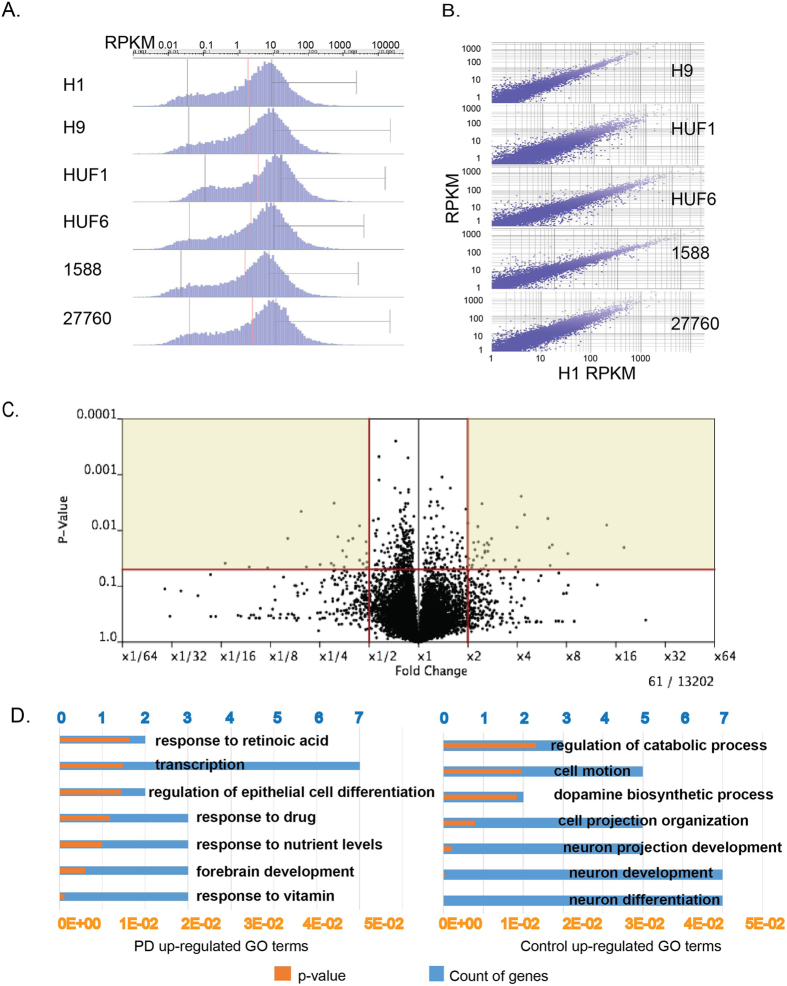
RNA-seq of human pluripotent stem cell derived dopaminergic neurons at day 50. (**A**) Histogram of transcripts of day 50 iDA neurons, Y-axis indicates number of genes expressed at a given RPKM (x-axis). (**B**) Scatter plots of gene expression patterns of H9, HUF1, HUF6, 1588 and 27760 against H1 derived iDA neurons. Pearson’s R processed signals of H9, HUF6, 1588 and 27760 are 0.97, 0.96, 0.96, 0.96, and 0.96 respectively. (**C**) Volcano plot of gene expression difference between control iDA neurons (H1, H9 and HUF1) and PD iDA neurons (HUF6, 1588, 27760). X-axis is fold change of gene expression (FPKM) of iDA _**hESC**_ / iDA _**iPSC**_. Y-axis indicates p-value. (**D**) GO analysis of differentially expressed genes (DEG), blue bar is number of genes under indicated GO term, orange bar indicates p-value of the indicated GO term.

To obtain gene expression profiles of iDA neurons, we extracted mRNA from 2 million cells on day 50 of differentiation for each line. Samples were then reverse transcribed and amplified prior to high-throughput analysis. For RNA-seq, more than 5 × 10^7^ pairs of reads were obtained for each sample, of which 70% to 80% had at least one end mapped to the reference genome. In each sample, we were able to detect more than 10,000 genes, with a distribution of gene expression from 0 to 5000 FPKM (Fig. 2A). Gene expression profiles from different cell lines show similar patterns with high correlations. The Pearson’s R values of processed signals between H1 and H9, HUF1, HUF6, 1588 and 27760 are 0.97, 0.96, 0.96, 0.96 and 0.96, respectively (Fig. 2B). Although the overall gene expression patterns of the six samples were similar, we cataloged difference in terms of gene expression between control (H1, H9, and HUF1) and PD-iPSC (HUF6, 1588 and 27760) derived neurons. There are only in total 61 genes with more than 2 fold changes of gene expression (p-values < 0.05) between these two sets (Fig. 2C). Among these genes, 37 genes were overexpressed in control-PSCs-derived neurons and 24 were overexpressed in PD-iPSC-derived iDA neurons (Supplemental Table 1). However, only 17 significant genes are under the FDR of 0.2 (Supplemental Table 1). Genes highly expressed in control iDA neurons have formed one cluster: leucine-rich repeat proteins, and are enriched for GO terms such as “neuron development”, “dopamine biosynthetic process” and “neuron projection process” etc. (Fig. 2D, Supplemental Table 2). Genes more highly expressed in PD iDA neurons are enriched for GO terms such as “response to vitamin”, “response to nutrient levels” and “transcription” etc. (Fig. 2D). Notably, there are some important dopaminergic neuron specific genes such as TH, LMX1B, NR4A2 under-expressed in PD-iDA neurons.

### Gene expression of iDA neurons and mDA neurons

**Figure 3:**
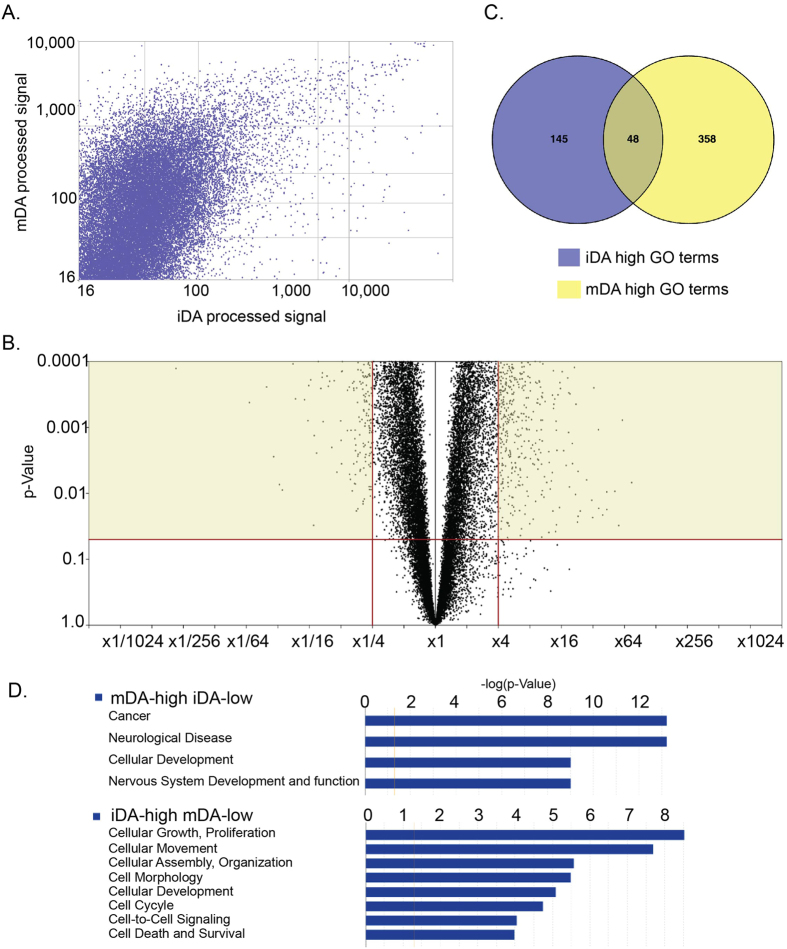
Compare gene expressions of induced DA neurons (iDA) and midbrain DA neurons (GSE7621). (**A**) Scatter plots of gene expression patterns of iDA against mDA, examining correlation of gene expression. Pearson’s R is 0.625. X-axis is log2 transformed microarray mean signal for mDA, y-axis is log2 transformed microarray mean signal for iDA. (**B**) Venn diagram of GO terms showing shared GO terms between mDA and iDA enriched terms. (**C**) Volcano plot of gene expression difference between control iDA neurons and mDA neurons (GSE7621). X-axis is fold change of gene expression of iDA/ mDA, Y-axis is p-value. (**D**) Ingenuity Pathway Analysis (IPA) of differentially expressed genes. Genes expressed high in mDA but low in iDA fall into gene ontology categories of cancer, neurological disease, cellular development and nervous system development; Genes expressed high in iDA but low in mDA fall into gene ontology categories of cellular growth and proliferation, cellular movement, cell assembly, cellular development, cell cycle, cell signaling and cell survival. X-axis is –log (p-value) of certain category, indicating significance of that category.

Previous results showed that iDA neurons mimic substantia nigra dopaminergic neurons in several aspects including dopamine release, electrophysiological features and *in vivo* function^17^. However, whether FP-derived iDA neurons resemble *substantia nigra pars compacta* (*SNpc*) DA neurons *in vivo,* in terms of its whole genome transcriptional profile has not been reported. Thus, we performed microarray analysis of iDA neurons and compared them to Papapetropoulos *et al.*’s (2006) published microarray data from human control and Parkinsonism *SNpc*^18^. Scatter plot analysis identified that the Pearson’s R-value between iDA and mDA is 0.658 (Fig. 3A), indicating that a significant difference exists between these two cell populations in terms of their transcriptome. Among differentially expressed genes, 472 genes are at least 4 fold higher in iDA neurons (Fig. 3B, Supplemental Table 2); however, significantly more genes, a total of 2,235 genes (Fig. 3B, Supplemental Table 2), are expressed at a higher levels in mDA neurons. Further, GO analysis of the differentially expressed genes using DAVID tools generated 406 terms (GO_BP_FAT) in mDA neurons and 193 terms (GO_BP_FAT) in iDA neurons. Among all GO terms in the two lists, 48 GO terms are in common (Fig. 3C, Supplemental table 3).

The existence of large gene expression differences between iDA neurons and human substantia nigra mDA suggests there may be a fundamental difference between mDA and iDA neurons in their developmental stages, a difference which is worth exploring. Thus, we turned to study the genes whose expressions are significantly altered. Firstly, we ranked genes in the two datasets according to their expression level within their groups. We extracted genes that are expressed at the top and bottom of each datasets (top or bottom 20%) to test if these genes are present in the opposite end in the other dataset (bottom or top 20%). After filtering, we generated two gene lists: iDA-high-mDA-low (162 genes, Supplemental Table 4) and iDA-low-mDA-high (65 genes, Supplemental Table 4). These two gene lists were then subjected to gene ontology analysis by IPA (Ingenuity Pathway Analysis, Qiagen), and were categorized into two distinct cellular function clusters: iDA-high genes were most closely related to cellular growth and proliferation, cellular movement, cell assembly, cellular development, cell cycle, cell signaling and cell survival (Fig. 3D). In contrast, iDA-low genes are more related to cancer, neurological disease, and nervous system development (Fig. 3D). The enrichment of cell cycle genes in iDA neurons and the lower levels of neurodevelopment genes raised the possibility that a large percentage of iDA neurons are still in an immature neuron stage that may be characterized by increased expression of cell proliferation genes.

**Figure 4:**
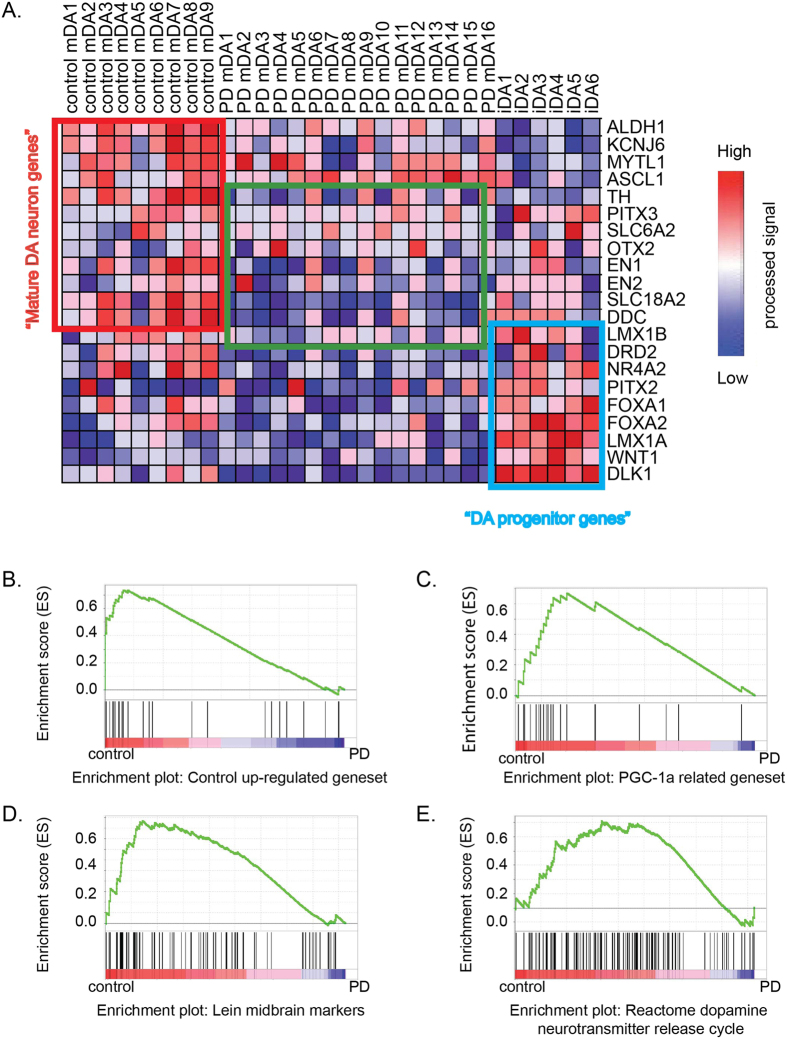
Gene Sets Enrichment Analysis (GSEA) of induced dopaminergic neurons for PD related features. (**A**) Heat map of important DA genes in all samples. Genes in red rectangle are genes highly expressed in mDA neurons, these genes include functional genes of mature DA neurons. Genes in the blue rectangle are genes highly expressed in iDA neurons, these genes include many progenitor markers. Genes in green rectangle show genes with reduced expression levels in PD patients. (**B**) GESA analysis of the PD iDA under –expressed geneset over control and PD patients data from datasets GSE7621. (**C**) GSEA analysis of Peroxisome proliferator–activated receptor γ coactivator-1α (PGC-1α) related genes over control-iDA and PD-iDA RNA-seq data. (**C**) GESA analysis of the “Lein midbrain markers” genes over control and PD patients’ data from RNAseq data. (**D**) GESA analysis of the “Reactome dopamine neurotransmitter release cycle” genes over control and PD patients’ data from RNA-seq data.

During human and mouse brain development, genes of diverse molecular functions are required, including transcription factors, plasma membrane markers, and genes specific to different developmental stages of dopaminergic neurons. To test if iDA neurons possess gene expression patterns indicative of progenitor or mature stages, we further compared well-characterized genes in terms of their expression in iDA and mDA neurons (Supplemental Table 5). Again, genes expressed in the progenitor stage *in vivo*, such as DLK1, FOXA1, LMX1A, WNT1, and FOXA2, were highly expressed in iDA cells, but low in mDA cells (Fig. 4A). Genes that are more highly expressed in mature DA neurons, such as TH, DDC, ALDH1, SLC18A2, KCNJ6, NR4A2, PITX3 and SLC6A3, were highly expressed in control mDA cells, but with reduced expression rank iDA neurons and PD mDA neurons (Fig. 4A). Notably, several transcription factors expressed in mature DA neurons *in vivo*, such as EN1, EN2 and PITX3, were not expressed or expressed only at very low levels in iDA neurons; moreover, protein expression of these factors was also undetectable by immunostaining (Supplemental Fig. 1A). This result suggests again that iDA neurons at day 50 are immature relative to their counterparts in the brain.

### GSEA analysis of induced dopaminergic neurons for PD related features

PD-associated DA neuron cell death generally occurs late in life. Compared to aged neurons, iDA neurons are in infancy. Whether iDA neurons can manifest PD phenotypes *in vitro* has been a subject of debate: some have reported PD phenotypes in fibroblasts before reprogramming, others have reported phenotypes early in development and others still have reported phenotypes dependent on maturation^19^,^20^,^21^,^22^,^23^. To test transcriptionally whether iDA neurons are more similar to PD neurons or control neurons from normal human samples, we examined the differentially expressed genes found between control and PD iDA neurons and subjected them to GSEA (Gene Set Enrichment Analysis) analysis between data from human PD and control samples (data from GSE7621). Consistent with our RNAseq comparison of iDA neurons, we observed that the set of genes that are under-expressed in iDA neurons derived from PD patients are also under-expressed mDA neurons from PD patients. These findings were derived from a GSEA analysis of mDA neurons from control patients vs mDA neurons from PD patients, which found enrichment of the gene set in mDA neurons from control patients (normalized enrichment score, or NES, of 1.57, p = 0.0004 and FDR q-value of 0.01). Genes over-expressed in iDA neurons from PD patients are not enriched in mDA neurons from either control or PD patients (Fig. 4B, Supplemental Figure 2). This finding suggests the differentially expressed genes found in the *in vitro* iDA neuronal models are also significantly differentially expressed in *in vivo* PD human brain samples.

In addition to analysis of the differentially expressed genes found in iDAs, other gene sets, especially those that are found to be associated with PD *in vivo*, may provide hints regarding differences between iDA and mDA neurons. Thus, we examined gene sets described by Zheng *et al.*^24^, which compared 185 laser captured human dopaminergic neuron samples and discovered 10 gene sets with previously unknown associations with PD, including genes that are responsive to peroxisome proliferator–activated receptor γ coactivator-1α (PGC-1α) (which are higher in control than PD). We tested whether the expression of PGC-1α related gene sets (supplemental table 5) resembles control-iDA or PD-iDA by GSEA. Clearly, PGC-1α related gene sets are enriched in the control-iDA samples with an NES of 1.63, FDR q-value of 0.058 (Fig. 4C). This result suggests that iPSC-iDA models could also distinguish PD-related gene sets discovered in patient samples. To further explore this idea, we tested 2 more related genesets: *Lein Midbrain Markers* and *Reactome Dopamine Neurotransmitter Release Cycle*. The *Lein Midbrain Marker*s geneset was derived from the Allen Brain Atlas database describing top ranked genes specific to the mouse midbrain. Again, this geneset is significantly enriched in our control-iDA phenotype in contrast to PD-iDA phenotype (Fig. 4D). However, the *Reactome Dopamine Neurotransmitter Release Cycle* was not enriched in either control or PD populations. Taken together, although there is a vast difference in terms of gene expression between mDA and iDA, iDA neurons still manifest important PD features previously found in patient studies. Furthermore, iDA studies can provide us with new insights about PD in terms of gene expression differences between control and PD cell lines.

## Discussion

In conclusion, our study found that the FP method commonly used to produce iDA neurons generated from different hPSC origins show similar gene expression patterns. However, iDA neurons were substantially different from *in vivo* mDA neurons in terms of global gene expression, especially genes defining their maturation stages. Notably, several transcriptional pathways important in mature DA neuron function and survival *in vivo* are reduced in iDA neurons (e.g., TH, DCC, KCNJ6, SLC18A2, and SLC6A3). We also show that iDA neurons overexpressed several important genes related to immature neurons, with appropriate dorsal-ventral axis gene patterning (FOXA2, LMX1A), but incorrect anterior-posterior axis gene patterning (e.g., EN1, EN2 and PITX3). This might partially explain the substantial difference in global gene expression patterns between iDA and mDA. Another potential explanation for the vast differences might be to their different cell environments—*in vivo* vs. *in vitro*.

Another notable finding is that, among the 37 genes under-expressed in PD iDA samples, there are several important DA neuron markers such as TH, NURR1, LMX1B and leucine-rich repeat proteins including LRRC4C (GWAS studies show association with PD. Since the PD lines are derived from patients with known PD-associated genetic variants, it is possible that their genetics lead to altered gene expression patterns for several important genes through hitherto unknown mechanisms. An interesting question then rises, do these mutations affect gene expression programs and if so how? Thus, although the differences are small, they may be significant and merit further study. Furthermore, it may be possible that as these cells mature and age, expression changes may begin to accumulate, causing greater divergence between the PD and healthy mDA neurons.

Taken together, these results suggest that iDA neurons are developmentally immature compared to mDA neurons. Although current induction protocols are not optimal for production of mature iDA neurons, they still possess several PD related features (i.e. underexpressed PGC-1α pathway genes). This suggests that further refinement of DA neuron induction might be necessary to enable differentiation of authentic midbrain dopaminergic neurons from stem cells.

## Materials and Methods

### hESC culture and neural induction

Human embryonic stem cell (hESC) lines H9 (WA-09, XX, passages 35–45), H1 (WA-01, XY, passages 30–40), the PD-carrying iPSC lines HUF6 (XY, homozygous LRRK2, passages 20–30) 1588 (Glucocerebrosidase (GBA) mutation; passages 20-30) and 27760 (XY, SNCA triplication, passages 20–30) and the control iPSC line HUF1 (passages 20–30) were maintained on matrigel plates (BD) in mTESR-1 hESC medium as described previously^21^. DA neuron differentiation followed the previously reported floor plate method^17^ but with minor modifications. Essentially, cells were plated on matrigel plates and exposed to LDN193189 (100 nM, Stemgent), SB431542 (10 μM, Tocris), SAG (0.25 μM, Tocris), purmorphamine (2 μM, Stemgent), FGF8 (50 ng/ml, R&D) and CHIR99021 (CHIR; 3 μM, Stemgent). Cells were plated (3.6 × 10^4^ cells per cm^2^) and grown for 11 days on matrigel in knockout serum replacement medium (KSR) containing DMEM, 15% knockout serum replacement, 2 mM Glutamax and 10 μM β-mercaptoethanol. KSR medium was gradually shifted to N2 medium starting on day 5 of differentiation as described previously^17^. On day 11, media was changed to neurobasal/B27/Glutamax containing medium (NB/B27; Invitrogen) supplemented with CHIR (until day 13) and with BDNF (brain-derived neurotrophic factor, 10 ng/ml; R&D), ascorbic acid (0.2 mM, Sigma), GDNF (glial cell line-derived neurotrophic factor; 10 ng/ml; R&D), TGFβ3 (transforming growth factor type β3, 1 ng/ml; R&D), dibutyryl cAMP (0.1 mM; Sigma), and DAPT (10 μM; Tocris,) for 9 days. On day 20, cells were dissociated using accutase (Innovative Cell Technology) and replated at high density (2 × 10^5^ cells per cm^2^) on dishes pre-coated with polyornithine (PO; 15 μg/ml)/laminin (1 μg/ml)/fibronectin (2 μg/ml) in differentiation medium (NB/B27 + BDNF, ascorbic acid, GDNF, dbcAMP, TGFβ3 and DAPT) until day 50 for RNA isolation.

### RNA isolation, library preparation and sequencing

Total RNA was extracted using an RNeasy kit (Qiagen). mRNA was isolated using Dynabeads mRNA purification kit (Ambion). RNA quality was determined by Bioanalyzer 2100 (Aglilent). mRNA was fragmented and first strand cDNA was synthesized by using Superscript III reverse transcriptase (Invitrogen). After second strand synthesis by DNA pol I (NEB), then we fragment the DNA and ligate the adaptors. Afterwards, we size select the adaptor ligated DNA and PCR amplify the ligated DNA. The library was generated using NEB Next DNA library prep master mix for Illumina (NEB). DNA library samples were submitted to the Stanford Genomics Facility and 76-base paired-end high throughput sequencing was performed. All sequenced libraries were mapped to the human genome using TopHat and Cufflink^25^,^26^, with default parameter setup.

### Microarray analysis

The Affymetrix GeneChip 133 2.0 plus was used for RNA expression profiling of 6 iDA RNA samples. RNA quality was controlled by Bioanalyzer 2100 (Agilent, Palo Alto, USA). Cel files were imported into Expression Console (Affymetrix), then normalized with RMA method provided by the software.

### Immunostaining and antibodies

Cells were fixed with 4% paraformaldehyde for 30 min at room temperature, and then washed with 0.1% Tween-20/PBS, 5 min, twice at room temperature. Cells were permeablized with 1% TritonX/PBS for 30 minutes at room temperature and blocked with 4% donkey serum/PBS, 30 min, room temperature. The primary antibodies were diluted in blocking solution and incubated with cells overnight at 4 °C, and then the cells were washed with 0.1% Tween-20/PBS, 5 min, three times at room temperature. Secondary antibodies were diluted in PBS at 1:200 dilution, incubated with processed cells for 1 hour at room temperature, and then washed with 0.1% Tween-20/PBS, 5 min each, three times at room temperature. Counterstaining was accomplished with DAPI/PBS (1 ug/mL DAPI in PBS). Primary antibodies were LMX1A (Millipore, ab10533), FOXA2 (Millipore, ab4125), OTX2 (Millipore, ab9566), TH (Pel Freez, P40101), GIRK2 (Abcam, ab30738), Nurr1 (Santa Cruz, sc-990) and TUBB3 (Covance, MRB-435 P). Secondary antibodies were from donkey (Jackson ImmunoResearch laboratories).

### Differential expression analysis and Gene Ontology analysis

Differential expression was analyzed using SUBIO platform (Subio.jp), Benjamini-Hochberg FDR was calculated using edgeR. To compare data generated from different platforms (RNA-seq and microarray), we focused on gene expression correlation rather than absolute values. Scatter plots were used to compare data generated from different transciptome analysis platforms and aided delineation of the phenotypic differences between iDA and mDA neurons. Gene ontology analysis was performed using Ingenuity Pathways Analysis (IPA, Qiagen).

### David analysis

Differentially expressed genes (DEGs) that passed all of the described filtering criteria (including the fold change cut off) were entered into the Gene Functional Annotation Tool available at the DAVID website ( http://david.abcc.ncifcrf.gov/) using their official gene symbols. Gene ontology options GOTERM_BP_ALL and were selected and a functional annotation chart generated. A maximum p-value of 0.05 was chosen to select only significant categories.

### GSEA analysis

Gene Set Enrichment Analysis (GSEA, Broad Institute) was used to classify different gene sets. Default parameters were used. For iDA neuron data sets, genes were ranked according to their FKPM expression levels and then the ranking list was submitted to GSEA using the pre-ranked mode.

### Metabolite Extraction

Briefly, proteins were precipitated from 200 □L neuronal or hNSC media using a 5:1 dilution with acetone and incubated overnight at −80 °C. Samples were then centrifuged at 24,000 × g for 15 min at −4 °C. Upper phase was collected and subsequently dried and resuspended in 20 □media using a 5:1 dilutn onto liquid chromatography based mass spectrometer (LCMS).

### LCMS Based Metabolome Analysis

LCMS analysis was performed using a 1290 UPLC coupled to a 6538 UHD Accurate-Mass Q-TOF (Agilent Technologies). The system was operated in positive ionization mode. Vials containing extracted metabolites and standard mixtures (Dopamine hydrochloride, Sigma Life Sciences) kept at -80 °C prior to LCMS analysis. Metabolites were separated using a Cogent Diamond Hydride, 150 mm, (HILIC). In positive ionization mode, A = 0.1% formic acid in water B = 0.1% formic acid in acetonitrile. HILIC linear gradient was 5% A (0–2 min) – 50% A, with an injection volume of 1 ul. ESI conditions are described here. Data was visualized using MassHunter software package (Agilent Technologies) and data processing was done using XCMS software, dopamine characterization was done using the Metlin database.

## Additional Information

**How to cite this article**: Xia, N. *et al.* Transcriptional comparison of human induced and primary midbrain dopaminergic neurons. *Sci. Rep.* **6**, 20270; doi: 10.1038/srep20270 (2016).

## Supplementary Material

Supplementary Information
