# Supplementary material for: Transcriptional comparison of human induced and primary midbrain dopaminergic neurons
Source: Sci Rep. 2016 Feb 4;6:20270. doi: 10.1038/srep20270 (PMC4740755; doi:10.1038/srep20270)
Supplement: Supplementary Information [file srep20270-s1.pdf]

Transcriptional comparison of human induced and primary midbrain dopaminergic neurons

Ninuo Xia[1,]*, Pengbo Zhang[1,]*, Fang Fang[1,] *, Zhengyuan Wang[3], Megan Rothstein[2], Benjamin Angulo[1], Rosaria Chiang[1], James Taylor[3] & Renee A Reijo Pera[1,2,]**

[1]Department of Genetics; Department of Obstetrics and Gynecology; Institute for Stem Cell Biology and Regenerative Medicine, Stanford University, CA, USA

[2]Department of Cell Biology and Neurosciences, Montana State University, 207 Montana Hall, Bozeman, MT

[3]Genomic Medicine Division, Hematology Branch, NHLBI/NIH, MD 20850, USA

**Correspondence: Renee A. Reijo Pera (renee.reijopera@montana.edu)

Supplemental Materials:
Supplemental Figure 1: iDA neurons do not express mDA markers EN1 or PITX3
Supplemental Figure 2: GESA analysis of the PD iDA over–expressed geneset over control and PD patients data from datasets GSE7621.
Supplemental Table 1: PD and control –iDA differential expressed genes and their expression values
Supplemental Table 2: GO terms of PD and control –iDA differential expressed genes
Supplemental Table 3: GO terms of mDA and iDA differential expressed genes
Supplemental Table 4: Gene list of significantly differential expressed genes between mDAs and iDAs
Supplemental Table 5: Genesets used in the study

Supplemental Figure Legend:
Supplemental Figure 1. iDA neurons do not express mDA markers EN1 or PITX3.
A. At day 50 of *in vitro* differentiation, cells don't express mature dopaminergic neuron proteins EN1 or PITX3.
B. Relative Abundance of Dopamine in iDA Neurons compared to human neural stem cells (hNSC). Y-axis: relative abundance of dopamine (154.0863 m/z, H+).

C. Bar graph of percentage of TH/FOXA2 positive cells of all 6 lines.

Supplemental Figure 2. GESA analysis of the PD iDA over–expressed geneset over control and PD patients data from datasets GSE7621. Note there is no enrichment of this geneset in either phenotype.

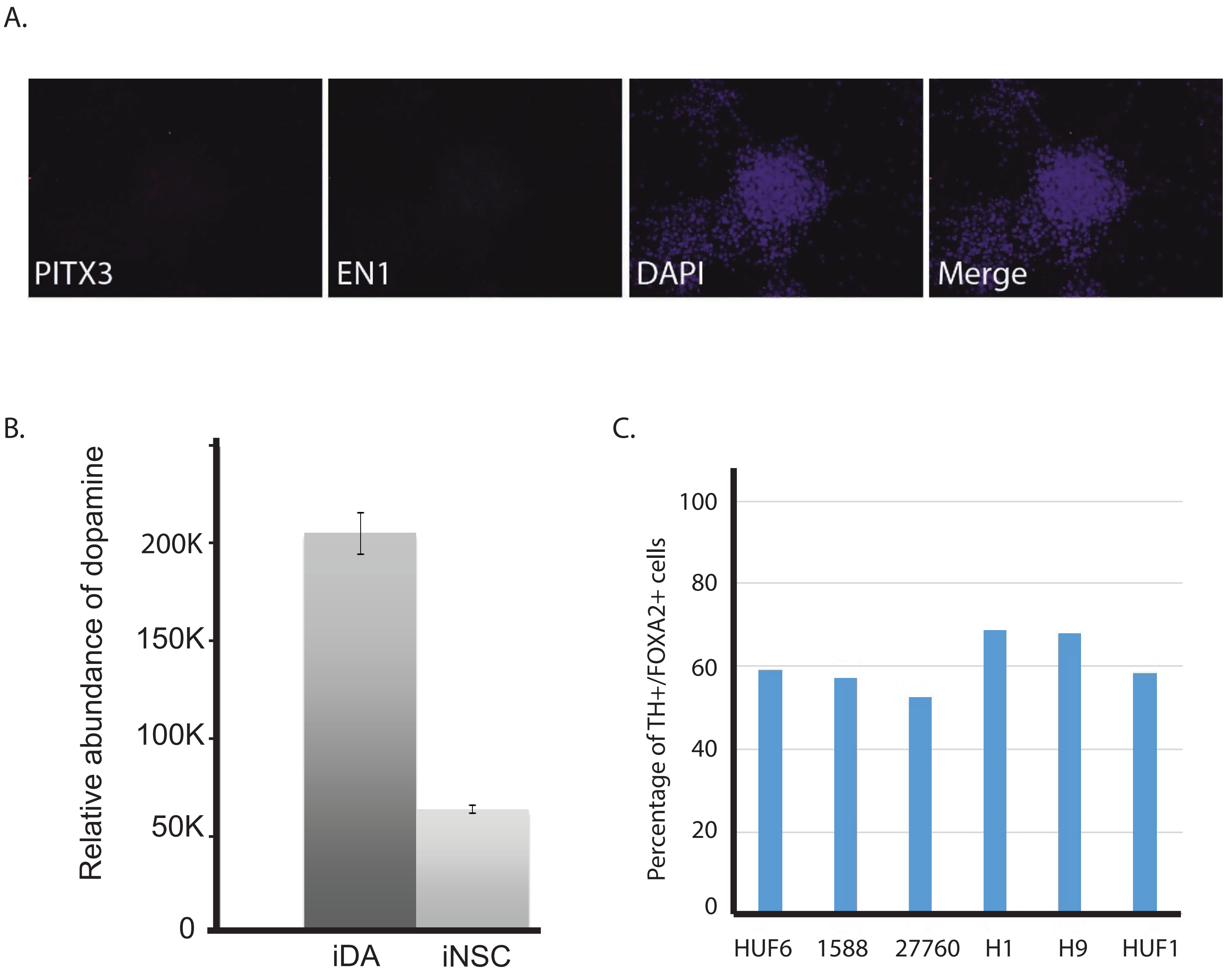

Xia et al. Supplementary Figure 1

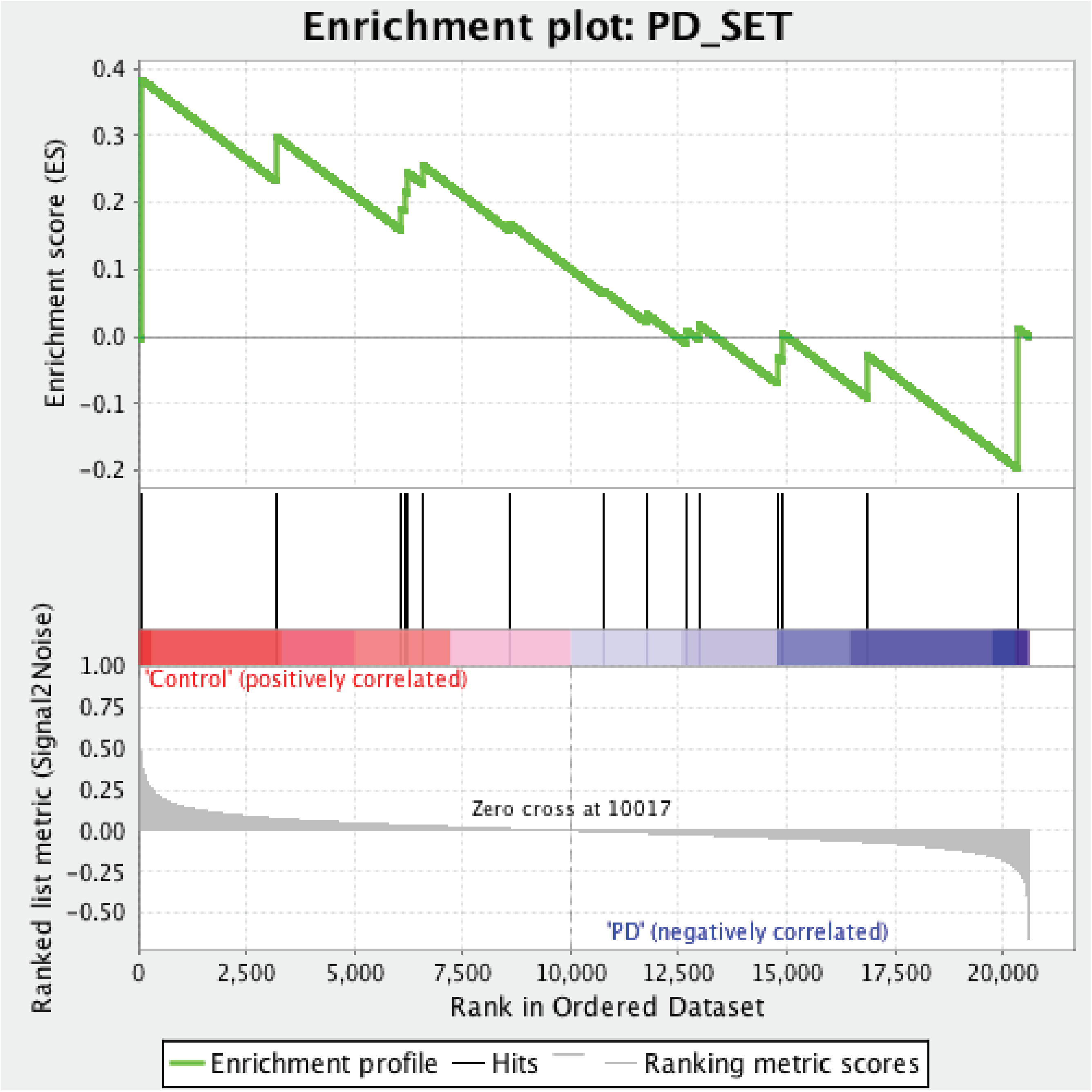

Xia et al. Supplementary Figure 2

Xia et al. Supplementary Table 1.

Genes up-regulated in control samples	Ch1 Raw Signal H1,H9,HUF1 control	Ch1 Raw Signal HUF6,1588,27760 PD	BH FDR
ASPN	11.822108	1.4566457	0.134
TH	82.921715	29.758783	0.775
NR4A2	21.358147	6.866361	0.364
BGN	13.609123	3.6781082	0.651
FOXB1	9.695494	4.093714	0.084
GRIN3A	13.281004	3.9298923	0.999
RBMS3	6.8861322	2.555585	0.234
F2RL2	5.5559025	1.3339295	0.152
SHISA6	13.426582	3.495181	0.712
LMX1B	29.099691	10.426176	0.193
PCDHB16	8.592311	1.5251385	0.219
PCSK1	463.65616	161.79248	0.234
CTHRC1	9.206776	2.9115112	0.512
LOC653513	7.8351626	3.1300733	0.330
IGFBP3	21.956505	2.1896539	0.193
CA10	15.68205	5.416114	0.999
CNTN4	31.935808	3.4706657	0.219
CHCHD2	89.23562	6.402126	0.134
NEDD4	2.7800558	0.8999299	0.999
SLITRK2	5.204939	0.12273729	0.134
LRRC4C	12.855011	4.883941	0.134
C9orf129	3.433015	0.9853961	0.999
TRIL	11.115684	3.2308497	0.775
KCTD8	10.69156	4.1506743	0.234
MYL9	5.203894	1.4023836	0.219
RPS132C	12.713156	0.17417082	0.134
APOC1	2.4146414	0.5856354	0.134
RIMBP2	22.387207	6.8331676	0.300
GRM7	16.562416	2.7731473	0.134
DPP6	26.898205	1.3617791	0.007
PKNOX2	15.913127	5.6736383	0.652
PDGFRB	2.7228484	0.47698563	0.999
DRP2	3.4064374	1.4340636	0.999
HMGA2	5.7932963	1.905998	0.999
RAMP2	32.6447	12.378548	0.775
KHDRBS2	6.896108	2.8608143	0.999
TIMP3	15.508622	3.0092835	0.134

Genes up-regulated in PD samples	Ch1 Raw Signal H1,H9,HUF1 control	Ch1 Raw Signal HUF6,1588,27760 PD	BH-FDR
FRG1B	2.3032148	13.391763	0.999
SEMA5B	4.0485563	10.298729	0.999
ARX	7.596926	14.186464	0.153
SOX21	2.3316584	9.583911	0.134
TCEAL5	1.3711429	22.011734	0.134
PUS7L	0.35393107	2.8772058	0.999
C21orf119	0.5390359	1.8150907	0.775
APLNR	3.5811155	30.052961	0.775
HSPB6	1.487791	3.0865393	0.999
ZNF528	0.860567	2.863408	0.999
RPL21P28	0.90301776	2.7515078	0.324
GPM6B	90.23138	195.56595	0.699
LRP2	1.92226	6.1526423	0.999
APOLD1	0.8296608	2.055738	0.999
NDP	0.90465146	2.5257082	0.733
BCHE	4.4772806	8.209116	0.775
C2orf40	1.0667772	4.3010006	0.735
SOX2	23.4999	61.37353	0.064
C17orf51	1.1062745	3.4589062	0.777
NME1-NME2	3.846185	6.6816444	0.999
INSM1	14.044105	20.741915	0.334
PCDHGB4	10.016014	37.83209	0.324
CTSF	0	5.319441	0.064
DNAJA4	0.447741	5.769682	0.134

Xia et al. Supplementary Table 2.

GO terms: PD down-regulated genes:

Category	Term	Count	%	PValue	List	Total	Pop Hits	Pop Total	Fold	Enrichment	FDR
GOTERM_BP_FAT	GO:0048666~neuron development	7	18.91891892	3.57E-05	27	339	13528	10.3459	0.020486	0.020486	0.05245
GOTERM_BP_FAT	GO:0030182~neuron differentiation	7	18.91891892	1.48E-04	27	438	13528	8.007441	0.082079	0.041918	0.216839
GOTERM_BP_FAT	GO:0031175~neuron projection development	5	13.51351351	0.00135	27	256	13528	9.78588	0.542951	0.22971	1.964946
GOTERM_BP_FAT	GO:0030030~cell projection organization	5	13.51351351	0.00501	27	368	13528	6.807568	0.945814	0.517528	7.122817
GOTERM_BP_FAT	GO:0042416~dopamine biosynthetic process	2	5.405405405	0.01148	27	6	13528	167.0123	0.998764	0.737946	15.60971
GOTERM_BP_FAT	GO:0006928~cell motion	5	13.51351351	0.01215	27	475	13528	5.274074	0.999167	0.69326	16.44932
GOTERM_BP_FAT	GO:0009894~regulation of catabolic process	3	8.108108108	0.0145	27	96	13528	15.65741	0.999791	0.701843	19.32208
GOTERM_BP_FAT	GO:0016477~cell migration	4	10.81081081	0.01544	27	276	13528	7.261406	0.99988	0.676377	20.44794
GOTERM_BP_FAT	GO:0051674~localization of cell	4	10.81081081	0.02046	27	307	13528	6.52817	0.999994	0.736028	26.20123
GOTERM_BP_FAT	GO:0048870~cell motility	4	10.81081081	0.02046	27	307	13528	6.52817	0.999994	0.736028	26.20123
GOTERM_BP_FAT	GO:0042423~catecholamine biosynthetic process	2	5.405405405	0.02095	27	11	13528	91.09764	0.999995	0.707073	26.74377
GOTERM_BP_FAT	GO:0033238~regulation of cellular amine metabolic process	2	5.405405405	0.02471	27	13	13528	77.08262	1	0.732666	30.77563
GOTERM_BP_FAT	GO:0051051~negative regulation of transport	3	8.108108108	0.02748	27	135	13528	11.13416	1	0.739873	33.6063
GOTERM_BP_FAT	GO:0031330~negative regulation of cellular catabolic process	2	5.405405405	0.03777	27	20	13528	50.1037	1	0.820543	43.22189
GOTERM_BP_FAT	GO:0042417~dopamine metabolic process	2	5.405405405	0.03777	27	20	13528	50.1037	1	0.820543	43.22189
GOTERM_BP_FAT	GO:0043062~extracellular structure organization	3	8.108108108	0.03882	27	163	13528	9.221541	1	0.806059	44.123
GOTERM_BP_FAT	GO:0009719~response to endogenous stimulus	4	10.81081081	0.0416	27	405	13528	4.948514	1	0.806612	46.45651
GOTERM_BP_FAT	GO:0010033~response to organic substance	5	13.51351351	0.04703	27	721	13528	3.474598	1	0.825589	50.74725
GOTERM_BP_FAT	GO:0048545~response to steroid hormone stimulus	3	8.108108108	0.05212	27	192	13528	7.828704	1	0.838985	54.47474
GOTERM_BP_FAT	GO:0007409~axonogenesis	3	8.108108108	0.05261	27	193	13528	7.78814	1	0.824704	54.81573
GOTERM_BP_FAT	GO:0042401~biogenic amine biosynthetic process	2	5.405405405	0.05614	27	30	13528	33.40247	1	0.828587	57.23044
GOTERM_BP_FAT	GO:0060284~regulation of cell development	3	8.108108108	0.05855	27	205	13528	7.332249	1	0.826158	58.8075
GOTERM_BP_FAT	GO:0010035~response to inorganic substance	3	8.108108108	0.05855	27	205	13528	7.332249	1	0.826158	58.8075
GOTERM_BP_FAT	GO:0007610~behavior	4	10.81081081	0.05967	27	469	13528	4.273237	1	0.817205	59.52681
GOTERM_BP_FAT	GO:0021954~central nervous system neuron development	2	5.405405405	0.05977	27	32	13528	31.31481	1	0.803057	59.5876
GOTERM_BP_FAT	GO:0048667~cell morphogenesis involved in neuron differentiation	3	8.108108108	0.06058	27	209	13528	7.191919	1	0.793171	60.09484
GOTERM_BP_FAT	GO:0048812~neuron projection morphogenesis	3	8.108108108	0.06263	27	213	13528	7.05686	1	0.790523	61.35944
GOTERM_BP_FAT	GO:0009712~catechol metabolic process	2	5.405405405	0.06339	27	34	13528	29.47277	1	0.781144	61.81518
GOTERM_BP_FAT	GO:0034311~diol metabolic process	2	5.405405405	0.06339	27	34	13528	29.47277	1	0.781144	61.81518
GOTERM_BP_FAT	GO:0006584~catecholamine metabolic process	2	5.405405405	0.06339	27	34	13528	29.47277	1	0.781144	61.81518
GOTERM_BP_FAT	GO:0018958~phenol metabolic process	2	5.405405405	0.0652	27	35	13528	28.63069	1	0.777745	62.8826
GOTERM_BP_FAT	GO:0009991~response to extracellular stimulus	3	8.108108108	0.06629	27	220	13528	6.832323	1	0.77085	63.51607
GOTERM_BP_FAT	GO:0009895~negative regulation of catabolic process	2	5.405405405	0.06879	27	37	13528	27.08308	1	0.771545	64.929
GOTERM_BP_FAT	GO:0007613~memory	2	5.405405405	0.07238	27	39	13528	25.69421	1	0.777465	66.86285
GOTERM_BP_FAT	GO:0014075~response to amine stimulus	2	5.405405405	0.07238	27	39	13528	25.69421	1	0.777465	66.86285
GOTERM_BP_FAT	GO:0021953~central nervous system neuron differentiation	2	5.405405405	0.07417	27	40	13528	25.05185	1	0.7746	67.78951
GOTERM_BP_FAT	GO:0000904~cell morphogenesis involved in differentiation	3	8.108108108	0.07934	27	244	13528	6.160291	1	0.787056	70.33804
GOTERM_BP_FAT	GO:0048858~cell projection morphogenesis	3	8.108108108	0.07991	27	245	13528	6.135147	1	0.778965	70.60251
GOTERM_BP_FAT	GO:0060341~regulation of cellular localization	3	8.108108108	0.0816	27	248	13528	6.060932	1	0.775976	71.38631
GOTERM_BP_FAT	GO:0032990~cell part morphogenesis	3	8.108108108	0.08616	27	256	13528	5.871528	1	0.784961	73.40581
GOTERM_BP_FAT	GO:0042176~regulation of protein catabolic process	2	5.405405405	0.09537	27	52	13528	19.27066	1	0.810031	77.08543
GOTERM_BP_FAT	GO:0007626~locomotory behavior	3	8.108108108	0.0967	27	274	13528	5.485807	1	0.805723	77.57588
GOTERM_BP_FAT	GO:0006898~receptor-mediated endocytosis	2	5.405405405	0.09711	27	53	13528	18.90706	1	0.798383	77.72683
GOTERM_BP_FAT	GO:0042398~cellular amino acid derivative biosynthetic process	2	5.405405405	0.09885	27	54	13528	18.55693	1	0.795814	78.35033

GO terms: PD up-regulated genes:

Category	Term	Count	%	PValue	List	Total	Pop Hits	Pop Total	Fold	Enrichment	FDR
GOTERM_BP_FAT	GO:0033273~response to vitamin	3	12.5	0.00304	18	66	13528	34.16162	0.654567	0.654567	4.0783
GOTERM_BP_FAT	GO:0007584~response to nutrient	3	12.5	0.01306	18	140	13528	16.10476	0.989837	0.899187	16.45281
GOTERM_BP_FAT	GO:0030900~forebrain development	3	12.5	0.01527	18	152	13528	14.83333	0.99535	0.833089	18.97298
GOTERM_BP_FAT	GO:0031667~response to nutrient levels	3	12.5	0.02486	18	197	13528	11.44501	0.999847	0.888762	29.11412
GOTERM_BP_FAT	GO:0042493~response to drug	3	12.5	0.02948	18	216	13528	10.43827	0.999971	0.876157	33.57515
GOTERM_BP_FAT	GO:0009991~response to extracellular stimulus	3	12.5	0.0305	18	220	13528	10.24848	0.99998	0.834946	34.51863
GOTERM_BP_FAT	GO:0030856~regulation of epithelial cell differentiation	2	8.33333333	0.03585	18	29	13528	51.83142	0.999997	0.837969	39.28871
GOTERM_BP_FAT	GO:0006350~transcription	7	29.1666667	0.03706	18	2101	13528	2.503993	0.999998	0.80743	40.32286
GOTERM_BP_FAT	GO:0021987~cerebral cortex development	2	8.33333333	0.04069	18	33	13528	45.54882	0.999999	0.800315	43.33185
GOTERM_BP_FAT	GO:0032526~response to retinoic acid	2	8.33333333	0.04069	18	33	13528	45.54882	0.999999	0.800315	43.33185
GOTERM_BP_FAT	GO:0021536~diencephalon development	2	8.33333333	0.04552	18	37	13528	40.62462	1	0.803266	47.10682
GOTERM_BP_FAT	GO:0033189~response to vitamin A	2	8.33333333	0.05152	18	42	13528	35.78836	1	0.81328	51.47528
GOTERM_BP_FAT	GO:0021543~pallium development	2	8.33333333	0.05629	18	46	13528	32.67633	1	0.814566	54.70985
GOTERM_BP_FAT	GO:0009628~response to abiotic stimulus	3	12.5	0.07673	18	368	13528	6.126812	1	0.882707	66.42307
GOTERM_BP_FAT	GO:0021537~telencephalon development	2	8.33333333	0.07866	18	65	13528	23.12479	1	0.870276	67.37312
GOTERM_BP_FAT	GO:0035270~endocrine system development	2	8.33333333	0.08331	18	69	13528	21.78422	1	0.867847	69.55156
GOTERM_BP_FAT	GO:0045449~regulation of transcription	7	29.1666667	0.09047	18	2601	13528	2.022641	1	0.873629	69.55156

Xia et al. Supplementary Table 3.

**145 elements included exclusively in "iDA upregulated genes":**
GO:0035270~endocrine system development
GO:0007242~intracellular signaling cascade
GO:0048534~hemopoietic or lymphoid organ development
GO:0030097~hemopoiesis
GO:0009719~response to endogenous stimulus
GO:0032147~activation of protein kinase activity
GO:0002520~immune system development
GO:0030900~forebrain development
GO:0009725~response to hormone stimulus
GO:0007267~cell-cell signaling
GO:0051952~regulation of amine transport
GO:0014070~response to organic cyclic substance
GO:0001889~liver development
GO:0031016~pancreas development
GO:0050433~regulation of catecholamine secretion
GO:0030098~lymphocyte differentiation
GO:0019935~cyclic-nucleotide-mediated signaling
GO:0045321~leukocyte activation
GO:0002521~leukocyte differentiation
GO:0043473~pigmentation
GO:0016055~Wnt receptor signaling pathway
GO:0006479~protein amino acid methylation
GO:0008213~protein amino acid alkylation
GO:0060341~regulation of cellular localization
GO:0048538~thymus development
GO:0001775~cell activation
GO:0030217~T cell differentiation
GO:0046649~lymphocyte activation
GO:0033674~positive regulation of kinase activity
GO:0051046~regulation of secretion
GO:0019932~second-messenger-mediated signaling
GO:0030858~positive regulation of epithelial cell differentiation
GO:0007618~mating
GO:0051347~positive regulation of transferase activity
GO:0040017~positive regulation of locomotion
GO:0007507~heart development
GO:0030856~regulation of epithelial cell differentiation
GO:0042493~response to drug

GO:0008277~regulation of G-protein coupled receptor protein signaling pathway
GO:0042113~B cell activation
GO:0009798~axis specification
GO:0009755~hormone-mediated signaling
GO:0051329~interphase of mitotic cell cycle
GO:0048706~embryonic skeletal system development
GO:0030003~cellular cation homeostasis
GO:0040012~regulation of locomotion
GO:0045860~positive regulation of protein kinase activity
GO:0032870~cellular response to hormone stimulus
GO:0006919~activation of caspase activity
GO:0043281~regulation of caspase activity
GO:0051325~interphase
GO:0043549~regulation of kinase activity
GO:0030005~cellular di-, tri-valent inorganic cation homeostasis
GO:0031667~response to nutrient levels
GO:0045665~negative regulation of neuron differentiation
GO:0051954~positive regulation of amine transport
GO:0051047~positive regulation of secretion
GO:0045165~cell fate commitment
GO:0030334~regulation of cell migration
GO:0006730~one-carbon metabolic process
GO:0048255~mRNA stabilization
GO:0043489~RNA stabilization
GO:0050885~neuromuscular process controlling balance
GO:0045216~cell-cell junction organization
GO:0003001~generation of a signal involved in cell-cell signaling
GO:0031960~response to corticosteroid stimulus
GO:0010952~positive regulation of peptidase activity
GO:0043280~positive regulation of caspase activity
GO:0002237~response to molecule of bacterial origin
GO:0007187~G-protein signaling, coupled to cyclic nucleotide second messenger
GO:0035113~embryonic appendage morphogenesis
GO:0030326~embryonic limb morphogenesis
GO:0055066~di-, tri-valent inorganic cation homeostasis
GO:0051338~regulation of transferase activity
GO:0002026~regulation of the force of heart contraction
GO:0043278~response to morphine
GO:0001959~regulation of cytokine-mediated signaling pathway
GO:0014072~response to isoquinoline alkaloid
GO:0050905~neuromuscular process
GO:0030335~positive regulation of cell migration
GO:0030512~negative regulation of transforming growth factor beta receptor signaling

pathway
GO:0060070~Wnt receptor signaling pathway through beta-catenin
GO:0008284~positive regulation of cell proliferation
GO:0045859~regulation of protein kinase activity
GO:0051640~organelle localization
GO:0007492~endoderm development
GO:0007043~cell-cell junction assembly
GO:0010604~positive regulation of macromolecule metabolic process
GO:0010551~regulation of specific transcription from RNA polymerase II promoter
GO:0003013~circulatory system process
GO:0008015~blood circulation
GO:0018125~peptidyl-cysteine methylation
GO:0033630~positive regulation of cell adhesion mediated by integrin
GO:0001961~positive regulation of cytokine-mediated signaling pathway
GO:0030219~megakaryocyte differentiation
GO:0032922~circadian regulation of gene expression
GO:0055080~cation homeostasis
GO:0009991~response to extracellular stimulus
GO:0035295~tube development
GO:0045761~regulation of adenylate cyclase activity
GO:0042110~T cell activation
GO:0042594~response to starvation
GO:0009968~negative regulation of signal transduction
GO:0051050~positive regulation of transport
GO:0043065~positive regulation of apoptosis
GO:0051656~establishment of organelle localization
GO:0043414~biopolymer methylation
GO:0007631~feeding behavior
GO:0048592~eye morphogenesis
GO:0051272~positive regulation of cell motion
GO:0001825~blastocyst formation
GO:0043068~positive regulation of programmed cell death
GO:0048545~response to steroid hormone stimulus
GO:0006357~regulation of transcription from RNA polymerase II promoter
GO:0009617~response to bacterium
GO:0051270~regulation of cell motion
GO:0031279~regulation of cyclase activity
GO:0035107~appendage morphogenesis
GO:0035108~limb morphogenesis
GO:0010942~positive regulation of cell death
GO:0048593~camera-type eye morphogenesis
GO:0008217~regulation of blood pressure
GO:0002064~epithelial cell development

GO:0008038~neuron recognition
GO:0051339~regulation of lyase activity
GO:0030817~regulation of cAMP biosynthetic process
GO:0006937~regulation of muscle contraction
GO:0006875~cellular metal ion homeostasis
GO:0051241~negative regulation of multicellular organismal process
GO:0007035~vacuolar acidification
GO:0043589~skin morphogenesis
GO:0016198~axon choice point recognition
GO:0030111~regulation of Wnt receptor signaling pathway
GO:0030814~regulation of cAMP metabolic process
GO:0060173~limb development
GO:0048736~appendage development
GO:0043488~regulation of mRNA stability
GO:0045670~regulation of osteoclast differentiation
GO:0048732~gland development
GO:0009954~proximal/distal pattern formation
GO:0033059~cellular pigmentation
GO:0051174~regulation of phosphorus metabolic process
GO:0019220~regulation of phosphate metabolic process
GO:0030183~B cell differentiation
GO:0032259~methylation

358 elements included exclusively in "mDA up-regulated genes":
GO:0006091~generation of precursor metabolites and energy
GO:0006119~oxidative phosphorylation
GO:0045333~cellular respiration
GO:0022900~electron transport chain
GO:0022904~respiratory electron transport chain
GO:0042775~mitochondrial ATP synthesis coupled electron transport
GO:0042773~ATP synthesis coupled electron transport
GO:0015980~energy derivation by oxidation of organic compounds
GO:0006120~mitochondrial electron transport, NADH to ubiquinone
GO:0046907~intracellular transport
GO:0008380~RNA splicing
GO:0051129~negative regulation of cellular component organization
GO:0016071~mRNA metabolic process
GO:0000377~RNA splicing, via transesterification reactions with bulged adenosine as nucleophile
GO:0000375~RNA splicing, via transesterification reactions
GO:0000398~nuclear mRNA splicing, via spliceosome
GO:0006397~mRNA processing
GO:0006396~RNA processing

GO:0033043~regulation of organelle organization
GO:0070727~cellular macromolecule localization
GO:0034613~cellular protein localization
GO:0007010~cytoskeleton organization
GO:0070507~regulation of microtubule cytoskeleton organization
GO:0031110~regulation of microtubule polymerization or depolymerization
GO:0006979~response to oxidative stress
GO:0032886~regulation of microtubule-based process
GO:0006413~translational initiation
GO:0006886~intracellular protein transport
GO:0032269~negative regulation of cellular protein metabolic process
GO:0007026~negative regulation of microtubule depolymerization
GO:0031114~regulation of microtubule depolymerization
GO:0016044~membrane organization
GO:0010639~negative regulation of organelle organization
GO:0051248~negative regulation of protein metabolic process
GO:0006796~phosphate metabolic process
GO:0006793~phosphorus metabolic process
GO:0031400~negative regulation of protein modification process
GO:0006511~ubiquitin-dependent protein catabolic process
GO:0031111~negative regulation of microtubule polymerization or depolymerization
GO:0016310~phosphorylation
GO:0006605~protein targeting
GO:0030029~actin filament-based process
GO:0043933~macromolecular complex subunit organization
GO:0034599~cellular response to oxidative stress
GO:0030705~cytoskeleton-dependent intracellular transport
GO:0030036~actin cytoskeleton organization
GO:0010498~proteasomal protein catabolic process
GO:0043161~proteasomal ubiquitin-dependent protein catabolic process
GO:0009060~aerobic respiration
GO:0015986~ATP synthesis coupled proton transport
GO:0015985~energy coupled proton transport, down electrochemical gradient
GO:0034220~ion transmembrane transport
GO:0006754~ATP biosynthetic process
GO:0046034~ATP metabolic process
GO:0006446~regulation of translational initiation
GO:0051494~negative regulation of cytoskeleton organization
GO:0051443~positive regulation of ubiquitin-protein ligase activity
GO:0010970~microtubule-based transport
GO:0042274~ribosomal small subunit biogenesis
GO:0007272~ensheathment of neurons
GO:0008366~axon ensheathment

GO:0042552~myelination
GO:0009206~purine ribonucleoside triphosphate biosynthetic process
GO:0022604~regulation of cell morphogenesis
GO:0034614~cellular response to reactive oxygen species
GO:0032990~cell part morphogenesis
GO:0009145~purine nucleoside triphosphate biosynthetic process
GO:0009201~ribonucleoside triphosphate biosynthetic process
GO:0016265~death
GO:0051351~positive regulation of ligase activity
GO:0051437~positive regulation of ubiquitin-protein ligase activity during mitotic cell cycle
GO:0016192~vesicle-mediated transport
GO:0031344~regulation of cell projection organization
GO:0008219~cell death
GO:0009144~purine nucleoside triphosphate metabolic process
GO:0031396~regulation of protein ubiquitination
GO:0031398~positive regulation of protein ubiquitination
GO:0022613~ribonucleoprotein complex biogenesis
GO:0009152~purine ribonucleotide biosynthetic process
GO:0009205~purine ribonucleoside triphosphate metabolic process
GO:0031397~negative regulation of protein ubiquitination
GO:0033365~protein localization in organelle
GO:0019941~modification-dependent protein catabolic process
GO:0043632~modification-dependent macromolecule catabolic process
GO:0009199~ribonucleoside triphosphate metabolic process
GO:0051789~response to protein stimulus
GO:0009260~ribonucleotide biosynthetic process
GO:0009142~nucleoside triphosphate biosynthetic process
GO:0051436~negative regulation of ubiquitin-protein ligase activity during mitotic cell cycle
GO:0031145~anaphase-promoting complex-dependent proteasomal ubiquitin-dependent protein catabolic process
GO:0048812~neuron projection morphogenesis
GO:0009141~nucleoside triphosphate metabolic process
GO:0051439~regulation of ubiquitin-protein ligase activity during mitotic cell cycle
GO:0006461~protein complex assembly
GO:0070271~protein complex biogenesis
GO:0015992~proton transport
GO:0051603~proteolysis involved in cellular protein catabolic process
GO:0030163~protein catabolic process
GO:0051352~negative regulation of ligase activity
GO:0051444~negative regulation of ubiquitin-protein ligase activity
GO:0044257~cellular protein catabolic process
GO:0034622~cellular macromolecular complex assembly
GO:0051438~regulation of ubiquitin-protein ligase activity

GO:0000059~protein import into nucleus, docking
GO:0031175~neuron projection development
GO:0006818~hydrogen transport
GO:0044265~cellular macromolecule catabolic process
GO:0044092~negative regulation of molecular function
GO:0006457~protein folding
GO:0070647~protein modification by small protein conjugation or removal
GO:0019228~regulation of action potential in neuron
GO:0055114~oxidation reduction
GO:0034621~cellular macromolecular complex subunit organization
GO:0010975~regulation of neuron projection development
GO:0051340~regulation of ligase activity
GO:0008088~axon cargo transport
GO:0009150~purine ribonucleotide metabolic process
GO:0006986~response to unfolded protein
GO:0050808~synapse organization
GO:0007265~Ras protein signal transduction
GO:0048858~cell projection morphogenesis
GO:0009259~ribonucleotide metabolic process
GO:0000226~microtubule cytoskeleton organization
GO:0050770~regulation of axonogenesis
GO:0001508~regulation of action potential
GO:0012501~programmed cell death
GO:0016053~organic acid biosynthetic process
GO:0046394~carboxylic acid biosynthetic process
GO:0009057~macromolecule catabolic process
GO:0006892~post-Golgi vesicle-mediated transport
GO:0046324~regulation of glucose import
GO:0031399~regulation of protein modification process
GO:0043086~negative regulation of catalytic activity
GO:0010605~negative regulation of macromolecule metabolic process
GO:0006915~apoptosis
GO:0010827~regulation of glucose transport
GO:0007017~microtubule-based process
GO:0042743~hydrogen peroxide metabolic process
GO:0051170~nuclear import
GO:0051058~negative regulation of small GTPase mediated signal transduction
GO:0046580~negative regulation of Ras protein signal transduction
GO:0010769~regulation of cell morphogenesis involved in differentiation
GO:0006164~purine nucleotide biosynthetic process
GO:0044087~regulation of cellular component biogenesis
GO:0006163~purine nucleotide metabolic process
GO:0006122~mitochondrial electron transport, ubiquinol to cytochrome c

GO:0051260~protein homooligomerization
GO:0051259~protein oligomerization
GO:0007156~homophilic cell adhesion
GO:0017038~protein import
GO:0006800~oxygen and reactive oxygen species metabolic process
GO:0043254~regulation of protein complex assembly
GO:0046326~positive regulation of glucose import
GO:0010828~positive regulation of glucose transport
GO:0007155~cell adhesion
GO:0022610~biological adhesion
GO:0006606~protein import into nucleus
GO:0030258~lipid modification
GO:0022618~ribonucleoprotein complex assembly
GO:0000245~spliceosome assembly
GO:0042273~ribosomal large subunit biogenesis
GO:0006631~fatty acid metabolic process
GO:0046356~acetyl-CoA catabolic process
GO:0006099~tricarboxylic acid cycle
GO:0050804~regulation of synaptic transmission
GO:0007409~axonogenesis
GO:0006916~anti-apoptosis
GO:0007266~Rho protein signal transduction
GO:0034504~protein localization in nucleus
GO:0043623~cellular protein complex assembly
GO:0010324~membrane invagination
GO:0006897~endocytosis
GO:0048193~Golgi vesicle transport
GO:0008154~actin polymerization or depolymerization
GO:0015931~nucleobase, nucleoside, nucleotide and nucleic acid transport
GO:0032446~protein modification by small protein conjugation
GO:0001578~microtubule bundle formation
GO:0009448~gamma-aminobutyric acid metabolic process
GO:0001505~regulation of neurotransmitter levels
GO:0006928~cell motion
GO:0042692~muscle cell differentiation
GO:0006633~fatty acid biosynthetic process
GO:0042391~regulation of membrane potential
GO:0031644~regulation of neurological system process
GO:0045104~intermediate filament cytoskeleton organization
GO:0051969~regulation of transmission of nerve impulse
GO:0042254~ribosome biogenesis
GO:0043524~negative regulation of neuron apoptosis
GO:0032271~regulation of protein polymerization

GO:0032273~positive regulation of protein polymerization
GO:0007422~peripheral nervous system development
GO:0016601~Rac protein signal transduction
GO:0010466~negative regulation of peptidase activity
GO:0051187~cofactor catabolic process
GO:0006084~acetyl-CoA metabolic process
GO:0009109~coenzyme catabolic process
GO:0007005~mitochondrion organization
GO:0044271~nitrogen compound biosynthetic process
GO:0051346~negative regulation of hydrolase activity
GO:0042744~hydrogen peroxide catabolic process
GO:0045103~intermediate filament-based process
GO:0007015~actin filament organization
GO:0009136~purine nucleoside diphosphate biosynthetic process
GO:0006172~ADP biosynthetic process
GO:0008090~retrograde axon cargo transport
GO:0009188~ribonucleoside diphosphate biosynthetic process
GO:0009180~purine ribonucleoside diphosphate biosynthetic process
GO:0050657~nucleic acid transport
GO:0050658~RNA transport
GO:0051236~establishment of RNA localization
GO:0030100~regulation of endocytosis
GO:0051130~positive regulation of cellular component organization
GO:0006612~protein targeting to membrane
GO:0042135~neurotransmitter catabolic process
GO:0031345~negative regulation of cell projection organization
GO:0006635~fatty acid beta-oxidation
GO:0022402~cell cycle process
GO:0070301~cellular response to hydrogen peroxide
GO:0006364~rRNA processing
GO:0031646~positive regulation of neurological system process
GO:0051262~protein tetramerization
GO:0001701~in utero embryonic development
GO:0050806~positive regulation of synaptic transmission
GO:0034976~response to endoplasmic reticulum stress
GO:0031333~negative regulation of protein complex assembly
GO:0046164~alcohol catabolic process
GO:0070646~protein modification by small protein removal
GO:0018105~peptidyl-serine phosphorylation
GO:0007018~microtubule-based movement
GO:0007346~regulation of mitotic cell cycle
GO:0050803~regulation of synapse structure and activity
GO:0016197~endosome transport

GO:0048167~regulation of synaptic plasticity
GO:0031334~positive regulation of protein complex assembly
GO:0032956~regulation of actin cytoskeleton organization
GO:0031401~positive regulation of protein modification process
GO:0034329~cell junction assembly
GO:0001504~neurotransmitter uptake
GO:0031346~positive regulation of cell projection organization
GO:0016072~rRNA metabolic process
GO:0046031~ADP metabolic process
GO:0032148~activation of protein kinase B activity
GO:0043523~regulation of neuron apoptosis
GO:0051247~positive regulation of protein metabolic process
GO:0050771~negative regulation of axonogenesis
GO:0042771~DNA damage response, signal transduction by p53 class mediator resulting in induction of apoptosis
GO:0006913~nucleocytoplasmic transport
GO:0008360~regulation of cell shape
GO:0022406~membrane docking
GO:0051971~positive regulation of transmission of nerve impulse
GO:0032970~regulation of actin filament-based process
GO:0016579~protein deubiquitination
GO:0021782~glial cell development
GO:0030330~DNA damage response, signal transduction by p53 class mediator
GO:0032270~positive regulation of cellular protein metabolic process
GO:0051169~nuclear transport
GO:0007163~establishment or maintenance of cell polarity
GO:0006378~mRNA polyadenylation
GO:0043030~regulation of macrophage activation
GO:0070584~mitochondrion morphogenesis
GO:0030041~actin filament polymerization
GO:0009165~nucleotide biosynthetic process
GO:0030968~endoplasmic reticulum unfolded protein response
GO:0034620~cellular response to unfolded protein
GO:0043255~regulation of carbohydrate biosynthetic process
GO:0016567~protein ubiquitination
GO:0016337~cell-cell adhesion
GO:0034654~nucleobase, nucleoside, nucleotide and nucleic acid biosynthetic process
GO:0034404~nucleobase, nucleoside and nucleotide biosynthetic process
GO:0015800~acidic amino acid transport
GO:0015868~purine ribonucleotide transport
GO:0035067~negative regulation of histone acetylation
GO:0015865~purine nucleotide transport
GO:0030834~regulation of actin filament depolymerization

GO:0051146~striated muscle cell differentiation
GO:0019320~hexose catabolic process
GO:0009133~nucleoside diphosphate biosynthetic process
GO:0019430~removal of superoxide radicals
GO:0006998~nuclear envelope organization
GO:0045454~cell redox homeostasis
GO:0019395~fatty acid oxidation
GO:0034440~lipid oxidation
GO:0018209~peptidyl-serine modification
GO:0008652~cellular amino acid biosynthetic process
GO:0051693~actin filament capping
GO:0044057~regulation of system process
GO:0007229~integrin-mediated signaling pathway
GO:0006508~proteolysis
GO:0031116~positive regulation of microtubule polymerization
GO:0008105~asymmetric protein localization
GO:0014823~response to activity
GO:0048741~skeletal muscle fiber development
GO:0006997~nucleus organization
GO:0017148~negative regulation of translation
GO:0043038~amino acid activation
GO:0043039~tRNA aminoacylation
GO:0006418~tRNA aminoacylation for protein translation
GO:0046365~monosaccharide catabolic process
GO:0006518~peptide metabolic process
GO:0010001~glial cell differentiation
GO:0046777~protein amino acid autophosphorylation
GO:0006575~cellular amino acid derivative metabolic process
GO:0048638~regulation of developmental growth
GO:0006096~glycolysis
GO:0006984~ER-nuclear signaling pathway
GO:0019216~regulation of lipid metabolic process
GO:0018107~peptidyl-threonine phosphorylation
GO:0043154~negative regulation of caspase activity
GO:0010256~endomembrane organization
GO:0009135~purine nucleoside diphosphate metabolic process
GO:0060052~neurofilament cytoskeleton organization
GO:0043090~amino acid import
GO:0043092~L-amino acid import
GO:0009179~purine ribonucleoside diphosphate metabolic process
GO:0045664~regulation of neuron differentiation
GO:0051646~mitochondrion localization
GO:0031112~positive regulation of microtubule polymerization or depolymerization

GO:0031113~regulation of microtubule polymerization
GO:0051056~regulation of small GTPase mediated signal transduction
GO:0030835~negative regulation of actin filament depolymerization
GO:0030433~ER-associated protein catabolic process
GO:0009066~aspartate family amino acid metabolic process
GO:0009062~fatty acid catabolic process
GO:0033554~cellular response to stress
GO:0010564~regulation of cell cycle process
GO:0032869~cellular response to insulin stimulus
GO:0032288~myelin assembly
GO:0006862~nucleotide transport
GO:0006402~mRNA catabolic process
GO:0001824~blastocyst development
GO:0007041~lysosomal transport
GO:0051783~regulation of nuclear division
GO:0007088~regulation of mitosis
GO:0006839~mitochondrial transport
GO:0010962~regulation of glucan biosynthetic process
GO:0021795~cerebral cortex cell migration
GO:0007263~nitric oxide mediated signal transduction
GO:0032885~regulation of polysaccharide biosynthetic process
GO:0019884~antigen processing and presentation of exogenous antigen
GO:0005979~regulation of glycogen biosynthetic process
GO:0043631~RNA polyadenylation
GO:0015813~L-glutamate transport
GO:0009894~regulation of catabolic process
GO:0030832~regulation of actin filament length
GO:0045176~apical protein localization
GO:0015991~ATP hydrolysis coupled proton transport
GO:0015988~energy coupled proton transport, against electrochemical gradient
GO:0047496~vesicle transport along microtubule
GO:0030031~cell projection assembly
GO:0033673~negative regulation of kinase activity
GO:0034330~cell junction organization
GO:0010675~regulation of cellular carbohydrate metabolic process
GO:0007528~neuromuscular junction development
GO:0030516~regulation of axon extension
GO:0007006~mitochondrial membrane organization
GO:0007416~synaptogenesis
GO:0046677~response to antibiotic
GO:0051289~protein homotetramerization

48 common elements in "iDA upregulated genes" and "mDA upregulated genes":

GO:0010033~response to organic substance
GO:0007268~synaptic transmission
GO:0030182~neuron differentiation
GO:0019226~transmission of nerve impulse
GO:0006412~translation
GO:0043085~positive regulation of catalytic activity
GO:0044093~positive regulation of molecular function
GO:0019725~cellular homeostasis
GO:0010035~response to inorganic substance
GO:0000902~cell morphogenesis
GO:0008104~protein localization
GO:0042592~homeostatic process
GO:0055082~cellular chemical homeostasis
GO:0032989~cellular component morphogenesis
GO:0010608~posttranscriptional regulation of gene expression
GO:0007264~small GTPase mediated signal transduction
GO:0000278~mitotic cell cycle
GO:0048666~neuron development
GO:0006873~cellular ion homeostasis
GO:0006414~translational elongation
GO:0043244~regulation of protein complex disassembly
GO:0042981~regulation of apoptosis
GO:0043067~regulation of programmed cell death
GO:0010941~regulation of cell death
GO:0042542~response to hydrogen peroxide
GO:0052548~regulation of endopeptidase activity
GO:0052547~regulation of peptidase activity
GO:0015031~protein transport
GO:0032535~regulation of cellular component size
GO:0050801~ion homeostasis
GO:0048667~cell morphogenesis involved in neuron differentiation
GO:0045184~establishment of protein localization
GO:0000904~cell morphogenesis involved in differentiation
GO:0007049~cell cycle
GO:0043242~negative regulation of protein complex disassembly
GO:0043066~negative regulation of apoptosis
GO:0043069~negative regulation of programmed cell death
GO:0060548~negative regulation of cell death
GO:0045637~regulation of myeloid cell differentiation
GO:0010038~response to metal ion
GO:0006403~RNA localization

GO:0032268~regulation of cellular protein metabolic process
GO:0030030~cell projection organization
GO:0048878~chemical homeostasis
GO:0065003~macromolecular complex assembly
GO:0000302~response to reactive oxygen species
GO:0051493~regulation of cytoskeleton organization
GO:0006417~regulation of translation

Xia et al. Supplementary Table 4.

geneSymbol(top 20% in 5 cells and bottom 20% ni ctl SN cells)	geneSymbol (Top 20% in SN cells but bottom 20% in iDA cells)
PPP4R1	HBA1
C5orf13	HBB
KIAA1430	MOBP
GDF15	MIR1244-1
NCALD	CNDP1
ANXA2	MT1H
TRIO	EVI2A
C1QL1	SLC25A6
RBM39	MAG
KLHL23	OMG
HNRNPAB	HEPACAM
LRRC40	C11orf92
KIAA1967	MOG
DICER1	LOC220594
MLLT1	C3
FAM171A2	CMTM5
COX6A1	PCDHGA1
CDKN1C	NBPF11
MRPS26	RGPD4
MTSS1L	OPALIN
IGFBP5	SGK3
C16orf42	MT1M
PRKCZ	SLC6A3
CD46	VAMP7
SOX4	CD99
CD36	NTSR2
MATR3	MIR21
CALCA	FBXW12
KCNQ2	CTCFL
FBXO41	GJB6
SMARCD3	CX3CR1
RARS2	MRO
PPP1R9B	FOLH1
CTNNA1	C1QC
DR1	C10orf116
C1orf86	SRGN
ASCL1	RASL12
MAP2K2	VWF
MAP1LC3B2	LOC150622
LIMK1	C10orf128
CD276	ATP10B
CACNA1H	HSD11B1
TECR	HLA-G
NLGN2	P2RY12
SYT7	SLC5A11
UBE2D1	NINJ2
CGA	DNAH17
GADD45GIP1	GPIHBP1
CARS	FOLH1B

POLR2A
LMX1B
LRP1
MRPL12
RPPH1
ARHGAP33
EDARADD
KIAA1467
ANXA1
MFAP4
ATP8A1
TANC2
MIR622
SERINC2
GABBR1
DYNLRB1
UBE2D3
PTPN5
RPS9
AUTS2
ARHGEF12
CIB2
RNF144A
CASP3
GPR50
PLXNA1
MEIS3
ASNS
C9orf5
TXNRD1
ERGIC1
LPPR1
ZMIZ2
RBP1
PTPRA
TAPBP
CERCAM
MGAT5
LPAR2
C6orf174
YIPF2
KIAA0317
LITAF
PTPN1
CITED2
IGF2
MIDN
TFPI2
DGCR6L
SCYL1
EMID2
RHOA
MSI1
GPSM1
TAF15
CNR1
DPYSL4
EIF3F
LMX1A
TNKS
SLCO2B1
GJB1
LOC100272216
ABCG2
CSF1R
C1QB
ANKRD20A1
SLC39A12
HIST2H2AA3
ZBED1
AGAP11
DNAH3
C1QA
RBP7
LOC389831

YBX1
LGR4
MGAT3
EFNA2
GPC3
HNRNPH2
GOLM1
ECEL1
DACT3
HGSNAT
NR2F6
HMGB2
CRH
MMP14
FKBP10
COPE
UBE2B
COL1A1
PCBP2
DNAJB5
TPH1
C19orf6
GNRH2
MYEOV2
ARHGAP5
MTHFD2
SOX2
UTS2
FLJ22184
C14orf1
ST8SIA2
GABRA3
AGRN
DAZAP2
LOC283624
COMMD7
PCDHGB6
HSD17B4
ZNRF1
SLC6A17
PVRL1
GRIA2
MAP3K10
TBC1D16
PAICS
DCN
CACNG4
BTRC
COL6A1
C21orf59
MEX3A
PPIL4

Xia et al. Supplementary Table 5.

mDA important ge	PGC1a geneset	Control_set	Dopamine release	Midbrain markers	PD_set	KEGG PD
DLK1	CAMK2B	ASPN	RAB3A	AFAP1	PD	disease
LMX1A	CAMK1	TH	RIMS1	AGT	FRG1B	APAF1
LMX1B	PPP3CB	NR4A2	SLC18A2	AGTR2	SEMA5B	ATP5A1
FOXA2	CAMK1G	BGN	SNAP25	ALDOC	ARX	ATP5B
FOXA1	CAMK4	FOXB1	STX1A	AMIGO2	SOX21	ATP5C1
ASCL1	PPP3CC	GRIN3A	STXBP1	ANKRD55	TCEAL5	ATP5D
MYT1L	CAMK2D	RBMS3	SYN1	ARHGEF40	PUS7L	ATP5E
DAT	CAMK2G	F2RL2	SYN2	ARRDC1	C21orf119	ATP5F1
EN1	CALM3	SHISA6	SYN3	BAIAP3	APLNR	ATP5G1
EN2	PPP3CA	LMX1B	SYT1	BCAT1	HSPB6	ATP5G1P5
PITX3	MEF2A	PCDHB16	VAMP2	BNC2	ZNF528	ATP5G2
KCNJ6	SLC2A4	PCSK1		C17orf79	RPL21P28	ATP5G3
NR4A2	MEF2D	CTHRC1		CACHD1	GPM6B	ATP5H
PITX3	CAMK2A	LOC653513		CD83	LRP2	ATP5J
PITX2	PPARA	IGFBP3		CERS2	APOLD1	ATP5O
SLC6A2	YWHAH	CA10		CLGN	NDP	ATP6
DDC	HDAC5	CNTN4		COL15A1	BCHE	ATP8
OTX2	ESRRA	CHCHD2		CREBL2	C2orf40	CASP3
NGN2	PPARGC1A	NEDD4		DDC	SOX2	CASP9
TH	MEF2C	SLITRK2		DENND1B	C17orf51	COX1
VMAT2		LRRC4C		DLK1	NME1-NME2	COX2
SLC18A2		C9orf129		DNM3	INSM1	COX3
DRD2		TRIL		EBF3	PCDHGB4	COX4I1
ALDH1A1		KCTD8		ECE2	CTSF	COX4I2
WNT1		MYL9		EIF5A2	DNAJA4	COX5A
FGF8B		TMEM132C		EN1		COX5B
		APOC1		ENDOD1		COX6A1
		RIMBP2		EPHB1		COX6A2
		GRM7		GABRQ		COX6B1
		DPP6		GFRA1		COX6B2
		PKNOX2		GJE1		COX6C
		PDGFRB		GLRA1		COX6CP3
		DRP2		GLRA2		COX7A1
		HMGA2		GLRA3		COX7A2
		RAMP2		GLRA4		COX7A2L
		KHDRBS2		GPC5		COX7B
		TIMP3		GPR1		COX7B2
				GPR165P		COX7C
				HAP1		COX8A
				HMGA1		COX8C
				HTR2C		CYC1
				ITGA10		CYCS
				ITIH3		CYTB
				KANK4		GPR37
				KCTD9		HTRA2
				KIAA0930		LOC10013373
				KLHL1		LOC642502
				LRRC16B		LOC644310
				LTBP3		LOC727947
				MAGED2		LOC729317
				MTMR2		LRRK2
				NSMAF		ND1
				OPALIN		ND2
				P2RX5		ND3
				PANX2		ND4
				PCOLCE2		ND4L
				PCSK1N		ND5
				PHLDA3		ND6
				PIGT		NDUFA1
				PLP1		NDUFA10
				POU4F1		NDUFA2

				PRPH		NDUFA3
				PTGDS		NDUFA4
				QDPR		NDUFA4L2
				SEMA3F		NDUFA5
				SEMA4G		NDUFA6
				SLC17A6		NDUFA7
				SLC25A43		NDUFA8
				SLC39A14		NDUFA9
				SLC4A2		NDUFAB1
				SLC6A3		NDUFB1
				SLC7A3		NDUFB10
				SLC8A3		NDUFB2
				STEAP2		NDUFB3
				TCF7L2		NDUFB4
				TRAIP		NDUFB5
				TSC22D3		NDUFB6
				TTC39A		NDUFB7
				UBLCP1		NDUFB8
				VAT1L		NDUFB9
				ZFHX3		NDUFC1
				ZFHX4		NDUFC2
						NDUFS1
						NDUFS2
						NDUFS3
						NDUFS4
						NDUFS5
						NDUFS6
						NDUFS7
						NDUFS8
						NDUFV1
						NDUFV2
						NDUFV3
						PARK2
						PARK7
						PINK1
						PPID
						SDHA
						SDHB
						SDHC
						SDHD
						5-Sep
						SLC18A1
						SLC18A2
						SLC25A31
						SLC25A4
						SLC25A5
						SLC25A6
						SLC6A3
						SNCA
						SNCAIP
						TH
						UBA1
						UBA7
						UBB
						UBE2G1
						UBE2G2
						UBE2J1
						UBE2J2
						UBE2L3
						UBE2L6
						UCHL1
						UQCR10
						UQCR11
						UQCRB
						UQCRC1
						UQCRC2
						UQCRFS1
						UQCRH
						UQCRHL
						UQCRQ
						VDAC1
						VDAC2
						VDAC3
